# Erratum

**DOI:** 10.4269/ajtmh.16-0570err

**Published:** 2018-03

In *Am J Trop Med Hyg* 97(2): 403–406 by Kim et al (https://doi.org/10.4269/ajtmh.16-0570) there is a note that reads “Financial support: This study was supported by research funds from Chosun University Hospital 2016.” This note should be deleted.

